# The Compositional Aspects of Edible Flowers as an Emerging Horticultural Product

**DOI:** 10.3390/molecules26226940

**Published:** 2021-11-17

**Authors:** Eleomar de O. Pires, Francesco Di Gioia, Youssef Rouphael, Isabel C. F. R. Ferreira, Cristina Caleja, Lillian Barros, Spyridon A. Petropoulos

**Affiliations:** 1 Centro de Investigação de Montanha (CIMO), Instituto Politécnico de Bragança, Campus de Santa Apolónia, 5300-253 Bragança, Portugal; eleomar.junior@ipb.pt (E.d.O.P.J.); iferreira@ipb.pt (I.C.F.R.F.); ccaleja@ipb.pt (C.C.); 2 Department of Plant Science, The Pennsylvania State University, University Park, PA 16802, USA; fxd92@psu.edu; 3 Department of Agricultural Sciences, University of Naples Federico II, Via Universita 100, 80055 Portici, Italy; youssef.rouphael@unina.it; 4 Department of Agriculture Crop Production and Rural Environment, University of Thessaly, Fytokou Street, N. Ionia, 38446 Volos, Greece

**Keywords:** edible flowers, phenolic compounds, antioxidant activity, anthocyanins, alkaloids, betacyanins, pigments

## Abstract

Edible flowers are becoming very popular, as consumers are seeking healthier and more attractive food products that can improve their diet aesthetics and diversify their dietary sources of micronutrients. The great variety of flowers that can be eaten is also associated with high variability in chemical composition, especially in bioactive compounds content that may significantly contribute to human health. The advanced analytical techniques allowed us to reveal the chemical composition of edible flowers and identify new compounds and effects that were not known until recently. Considering the numerous species of edible flowers, the present review aims to categorize the various species depending on their chemical composition and also to present the main groups of compounds that are usually present in the species that are most commonly used for culinary purposes. Moreover, special attention is given to those species that contain potentially toxic or poisonous compounds as their integration in human diets should be carefully considered. In conclusion, the present review provides useful information regarding the chemical composition and the main groups of chemical compounds that are present in the flowers of the most common species.

## 1. Introduction

The use of flowers in human diet and culinary preparations is not something new but is dated back to the ancient years [1]. However, nowadays there is a growing trend in using flowers for edible purposes due to the increased awareness of consumers regarding the impact of diet on human health, as well as due to the revival of healthy lifestyles related to specific regions of the world, e.g., the Mediterranean diet [2,3]. Moreover, the high number of studies that explore the health benefits, the nutritional value, and the bioactive properties of edible flowers are the key drivers for the food industry and the consumers that demand the production of functional and healthy foods [4]. Apart from the apparent aesthetic effects of edible flowers in various dishes, the health aspects of their consumption is gaining more and more interest [5]. Therefore, integrating edible flowers in newly designed dishes and food formulations or increasing the consumers’ acceptability of traditional foods could help to increase the palette in human diet and diversify the dietary sources of nutrients and bioactive compounds on a daily basis [6,7]. This new approach also has a great impact on the so-called “culinary medicine” where food and cooking are blended with medicine and altogether contribute to the overall well-being [8].

The culinary flora includes several species whose flowers are edible and are commonly used for culinary purposes, including not only ornamental plants but also wild species, trees, and vegetables [9,10,11]. The high number of such species means that edible flowers contain a great variety of phytochemicals that contribute to their visual appearance (e.g., colorants such as anthocyanins, carotenoides) and bioactive properties (e.g., phenolic compounds) [9,12]. The popularity of these compounds is directly related to the numerous health benefits that their consumption may infer, especially their antioxidant potential and bioactivities, such as anti-inflammatory activity, cardioprotection, and prevention against some types of cancer [13]. Moreover, foods of natural origin that are rich in polyphenols are attractive for their positive impacts against chronic conditions such as obesity, diabetes, and neurodegenerative diseases [14]. However, apart from the positive effects on human health that edible flowers may have, there are several safety issues related to the presence of potential toxic or poisonous compounds and special attention should be given to novel species whose flowers are not commonly or traditionally used for culinary purposes [15]. Moreover, the lack of recommended daily allowances or reference daily intake suggestions and the variable composition of edible flowers should be carefully considered since irrational use of unsafe compounds could pose threats to human life [16].

Considering the variability in composition of edible flowers, the present review aims to present a detailed description of the chemical composition of edible flowers, focusing on those that have the most common uses in culinary preparations. The cited studies were retrieved after a thorough search in Scopus and Google Scholar databases, using keywords related to edible flowers or to species which are used for their flower parts.

## 2. Chemical Composition of Edible Flowers

**Figure 1:**
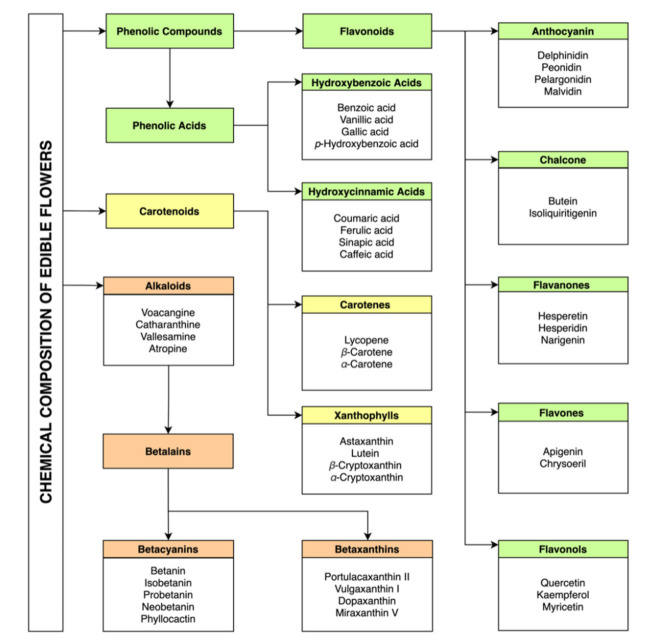
Examples of chemical compounds commonly found in edible flowers (authorship by the authors).

Edible flowers are sources of a wide variety of bioactive compounds, namely, phenolic compounds, carotenoids, betalains, and alkaloids. (Figure 1). There are several studies that identify and quantify the chemical profile of these compounds, as well as the association of their presence with several health benefits. The incorporation of different edible flowers in the diet, directly or through infusions, has been gaining more and more interest as it is recognized in having a positive action in reducing pathological symptoms [17,18]. This fact is directly linked to the phytochemical profile of flowers, due to the presence of bioactive compounds such as phenolic acids, flavonoids, alkaloids (mainly responsible for antioxidant activity) [19], and carotenoids (precursors of vitamin A which is responsible for vision health) [20].

The industrial sector has been testing various possibilities for introducing bioactive compounds from edible flowers in the formulation of new food products. This introduction has been tested by direct insertion of intact various floral parts (petals, stems, sepals) or by incorporating their extracts or essential oils [21]. Currently, there are several studies that report the functionality of flower phytochemicals when applied in different food formulations, namely in dairy products such as yogurt [22,23,24,25], ice cream [26], or pastry products [27,28,29].

According to Lu et al. [30], the main compounds present in edible flowers include the classes of carotenoids, phenolics, alkaloids, nitrogen-containing compounds and organosulfur compounds. Regarding the chemical composition, this can be very variable depending on the botanical part under analysis, e.g., carotenoids and flavonoids that can be found in pollen; inorganic ions, organic acids, phenolics, alkaloids and terpenoids detected in nectar; and vitamins, minerals and other compounds which are very frequently present in the petals [31]. In addition, there are several factors that may directly influence the chemical profile of flowers parts, such as the differences in color within cultivars of the same species, the soil and climate conditions, the production system, the flowering stage, or even the studied parts [32].

### 2.1. Phenolic Compounds

By definition, phenolic compounds include any substance with an aromatic ring, with one or more hydroxyl substituents [19]. As for their classification, they are organized according to the number of phenolic subunits present (polyphenols and simple phenols), defined as flavonoids and non-flavonoids and according to their structural arrangement [33]. They can be found in nature in free forms or conjugated with sugars, acids, and other biomolecules [13].

Phenolic compounds are directly involved in the growth, reproduction, and protection mechanisms of plants, besides promoting organoleptic aspects of color and flavor in their plant tissues [34]. They are present in fruits and flowers, comprising a wide range of aromatic secondary metabolites of plants, and they are capable of eliminating free radicals present in the human organism when ingested [35].

The presence of phenolic compounds in edible flowers has attracted the attention of the food industry, since they can be used as natural alternatives to artificial additives and food preservatives currently available on the market [17]. The identification and quantification of phenolic compounds present in edible flowers is extremely important for their inclusion in functional foods [19]. Among the numerous phenolic constituents existing in nature, phenolic acids (hydroxycinnamic and hydroxybenzoic acids) and flavonoids (anthocyanin, chalcone, flavanones, flavones, and flavonols) are highlighted as the majority and of high bioactive potential in edible flowers [19,36]. Considering the importance of phenolic compounds, the next sections will focus on the scientific research regarding the presence of the various classes of phenolic compounds in edible flowers.

#### 2.1.1. Phenolic Acids

Belonging to the family of phenolic compounds, phenolic acids are characterized by the presence of the phenolic function in their structural arrangement, being divided into the classes of hydroxycinnamic acids (C6-C3) and hydroxybenzoic acids (C6-C1) (Table 1), which represent the most important portion of non-flavonoid phenolic compounds [37].

Hydroxybenzoic acid derivatives have been detected in the edible flowers of various species, such as *Bougainvillea glabra* Choisy [38], *Abelmoschus manihot* (L.) Medik. [39]. While hydroxycinnamic acid has been reported in inflorescences of *Butea monosperma* (Lam.) Taub. and *Sesbania grandiflora* (L.) Pers. [40], and *Calendula officinalis* L. [41]. In a study on the chemical composition of 70 distinct species of Chinese edible flowers, Zheng, Meenu & Xu [19] identified several phenolic acids, in particular high contents of p-hydroxybenzoic acid in Cymbidium sinense (Jacks.) Willd. (1859.0 μg/g) *Canna indica* L. (1792.7 μg/g) *Magnolia denudata* Desr. (1743.4 μg/g), *Dianthus caryophyllus* L. (1398.5 μg/g) and *Calycanthus floridus* L. (1350.6 μg/g).

Fifteen phenolic acids were also identified in orange-colored specimens of Mexican Marigold (*Tagetes erecta* L.), in which hydroxybenzoic acids, namely, pyrogallol and gallic acid, were the most abundant phenolic acids in the flower extract (methanol and water (1:4)) [42]. The principal compounds detected in the ethanolic extract (75%) of *Hibiscus roseus* Thore flowers were mostly phenolic acid derivatives, namely p-Coumaric, chlorogenic, and trans-ferulic acids [43].

In the recent review study of Demasi et al. [44], the authors pointed out the presence of various phenolic acids in the edible flower of 26 different species, including benzoic (ellagic acid and gallic acid) and cinnamic acids (caffeic acid, chlorogenic acid, coumaric acid and ferulic acid) which stood out as the main derivatives of phenolic acids. In another study, the detected phenolic acids in floral extracts (ethanolic, 1:10, *v*/*v*) from 21 rose cultivars included gallic acid, p-coumaric acid, hesperidin and quercetin [45].

When investigating the influence of drying methods on the phenolic composition of chrysanthemum flowers, Lu et al. [46] identified five phenolic acid derivatives. In the same study, it was also noted that phenolic acids showed greater stability when compared to flavonoids after the dehydration process of the flowers. When studying the ethanolic extract (12%) from four distinct Brazilian edible flowers (*Amaranthus hypochondriacus* L., *Tropaeolum majus* L. (red), *Tropaeolum majus* L. (orange) and *Spilanthes oleracea* L.), p-coumaric and ferulic acids were reported for the first time in all the respective species investigated [47].

Four phenolic acids (gallic acid—1.05 ± 0.61 (mg/g); caffeic acid—0.19 ± 0.02 (mg/g); ferulic acid—0.48 ± 0.27 (mg/g); rosmarinic acid—5.49 ± 0.96 (mg/g)) were also identified in the flower extract of *Lavandula angustifolia* Mill., by ultrasound-assisted extraction with natural eutectic solvents (NADESs) and reference solvents (70% ethanol, 80% methanol) [48]. Krzymińska et al. [49], studied the phenolic acid profile of five cultivars of *Tulipa gesneriana* L., in which significant amounts of hydroxybenzoic acids (p-hydroxybenzoic, 2,5-dihydroxybenzoic, gallic, vanillic, syringic, salicylic, protocatechuic), and hydroxycinnamic acids (trans-cinnamic, p-coumaric, caffeic, ferulic, chlorogenic, sinapic) were identified, while it was noted that the profile of the phenolic acids of the flowers studied varied according to the cultivar, the production system, and the storage time.

#### 2.1.2. Flavonoids

The flavonoid family (commonly found in flowers) is subdivided into the classes of anthocyanins, chalcones, flavanones, flavones and flavonols [50], and comprises the largest group of secondary metabolites present in plants with about 15,000 different structures currently identified [51,52]. They are considered low molecular weight compounds and are recognized as non-nitrogenous plant pigments [53]. The antioxidant activity of flavonoids is directly related to their structural arrangement, due to the positions and amount of hydroxyl and methoxyl groups present in each specific compound [54]. They are regarded as the most significant natural phenolics, with the greatest diffusion and diversity of compounds present in the environment [20].

Flavonoids were observed in several edible flowers (Table 2), such as in samples of Calendula officinalis L., Ocimum sanctum L., Vinca rosea L., Hibiscus rosa-sinensis L. [41], Erythrina americana Mill., *Hibiscus sabdariffa* L., Opuntia ficus-indica (L.) Mill., Tagetes erecta L., Plumeria rubra L., Dahlia spp., Agave durangensis Gentry [55], and Artemisia spp. [56]. A recent study evaluated the phenolic profile of eleven edible flowers and highlighted Dianthus caryophyllus L. (17.50 mg/g), *Tagetes erecta L*. (16.90 mg/g) and Rosa hybrida L. (16.57 mg/g) as having the highest amount of total flavonoids detected [57].

Chen et al. [58] investigated the flavonoid composition of 23 different flowers and suggested that the species *Osmanthus fragrans* (Thunb.) Lour (71.49 mg/g), *Lavandula angustifolia* Mill (27.43 mg/g) and *Rosmarinus officinalis* L. (18.83 mg/g) showed the most promising results in terms of total flavonoids. Loizzo et al. [59], studied the flavonoids composition in flowers popularly consumed in Italy (*Anchusa azurea*, *Capparis spinosa*, *Cichorium intybus*, *Hedysarum coronarium*, *Malva sylvestris*, *Robinia pseudoacacia*, *Rosmarinus officinalis* and *Sambucus nigra*) and suggested rutin and quercitin as the main flavonoids.

Barriada-Bernal et al. [60], evaluated the composition of total flavonoids in ethanolic extract (60%, *v*/*v*) of dried flowers of *Agave duragenensis* L. and noted that the whole flowers were responsible for higher quantities of flavonoids (1210.4 µg/g of dry extract) when compared to extracts from individual parts (tepals, antars, and pollen). Moreover, in a study that included seventy species of Chinese edible flowers, the compounds quercitrin, hesperidin, quercetin, luteolin, kaempferol, hesperitin, and apigenin stood out as the main flavonoid compounds present [19].

##### Anthocyanins

Anthocyanins are natural pigments, capable of conferring attractive coloration on flowers in shades of orange, red, pink and blue [61,62], and although they have numerous derivatives in nature, the main components usually found are malvidin, petunidin, pelargonidin, peonidin, cyanidin and delphinidin [63,64]. They are related to the defense system of plants, being induced under conditions of abiotic stress (light and heat stress, water and nutrient deficit), biotic stress (attack by hebivores and microorganisms) or non-stress conditions (increase in leaf temperature, nutrient transport and regulation of osmotic balance) [65]. They are water-soluble compounds belonging to the flavonoid family, responsible for giving intense colors to fruits and flowers, while they are commonly used for culinary purposes as natural colorants [61,66,67,68,69]. There are many health benefits associated with the consumption of foods rich in anthocyanins, such as improvement in eye health, protection against cardiovascular diseases, anti-obesity and antidiabetic effects, and antimicrobial, anticancer or neuroprotective properties [70]. In terms of proportion, the anthocyanin content of flowers is directly related to their antioxidant potential [71].

Benvenuti et al. [71] revealed the presence of a high content of total anthocyanins highlighting cyanidin-3-*O*-glucoside (cyn-3-glu) as the major compound detected in red *Dianthus barbatus* L. (13.35 mg cyn-3-glu eq./100 g fw), blue *Viola wittrockiana* Gams (13.6 mg cyn-3-glu eq./100 g fw), and red *Petunia hybrida* Vilm. (14.44 mg cyn-3-glu eq./100 g fw). Analyzing the phenolic profile of *Tropaeolum majus* L. flowers, Garzón et al. [72] observed anthocyanin concentrations ranging from 31.9 ± 21.7 to 114.5 ± 2.3 mg of cyn-3-glu/100 g fw in yellow and red petals, respectively. The pigmentation of *Tropaeolum majus* L. flowers is in fact associated with the presence of specific anthocyanins, anthocyanin precursors, and carotenoids.

In turn, Hallmann [73] compared the phenolic content of pink (*Robinia hispida* L.) and white (*Robinia pseudoacacia* L.) flowers of the genus *Robinia* and she suggested the presence of antocyanins derivatives (cyanidin, pelargonidin, delphinidin, malvidin, and peonidin) in the pink colored flowers with cyanidin 3,5-di-*O*-glucoside and pelargonidin 3,5-di-*O*-glucoside as the main anthocyanins identified.

A study focused on the evaluation of anthocyanin content in hydroethanolic extracts of Sri Lankan edible flowers, revealed the presence of high amounts of cyanidin-3-*O*-glucoside and reported that the highest anthocyanin content was obtained from the extracts of *Hibiscus rosa-sinensis* L. (2003.5 ± 0.1 μg cyn-3-glu eq./g dw), *Ixora coccinea* L. (1573.1 ± 0.1 μg cyn-3-glu eq./g dw) and *Punica granatum* L. (1181.10 ± 0.02 μg cy-3-glucoside/g dw) flowers [41]. Anthocyanin derivatives were also reported by Yisimayili et al. [74] in *Punica granatum* L. flowers, which were further identified as delphinidin-3-*O*-glucoside, cyanidin-3,5-*O*-diglucoside, pelargonidin-3,5-*O*-diglucoside, cyanidin-3-*O*-glucoside, pelargonidin-3-*O*-glucoside.

Another study evaluating 70 edible flowers from China, highlighted cyanidin-3-*O*-glucoside as the predominant anthocyanin compound in *Bauhinia variegata* L. (10400.9 μg/g dw) and *Myosotis sylvatica* Hoffm. species (5208.9 μg/g dw), followed by malvidin-3-*O*-glucoside derivatives (most abundant in *Helichrysum bracteatum* (Venten.) Willd., 12,709.9 μg/g dw), peonidin-3-*O*-glucoside (most abundant in *Coreopsis tinctoria* Nutt., 1963.7 μg / g dw), cyanidin-3-*O*-sophoroside (the highest content detected in *Rosa chinensis* Jacq, 830.3 μg/g dw), pelargonidin-3-*O*-glucoside (the highest content detected in *Punica granatum* L., 1082.2 μg/g dw) and delphinidin-3-*O*-glucoside (the highest content detected in *Helichrysum bracteatum* (Venten.) Willd., 4721.6 μg/g dw) [19].

Chensom et al. [75] studied the anthocyanin content in the flowers of three different cultivars of *Titanbicus* (hybrids of *Hibiscus moscheutos* L. × *Hibiscus coccineus* Walt.), and reported the presence of cyanidin-3-glucoside and cyanidin-3-sambubioside. Moreover, the same authors demonstrated the high potential of these extracts as functional ingredients for food formulations, being effective in contributing to nutritional aspects and visual appearance [75]. Edible pumpkin flowers (*Cucurbita maxima* Duchesne) also presented relevant contents of anthocyanins, both in the methanolic extract (10.3 mg/100 g fw) and in aqueous extract (11.2 mg/100 g fw) [76].

A study performed by Sagdic et al. [77] presented ethanol extracts (ethanol/water, 1:1, *v*/*v*, acidified with 0.01% HCl) of four varieties (violet, red-orange, red claret, and rose) of *Tulipa* sp. L., as promising natural colorants with high potential for industrial application. The same suggestion was made by Pires et al. [78], who highlighted the anthocyanin extracts from *Impatiens walleriana* flowers as a promising colorant of orange and pink shades.

In a recent study, Barani et al. [79] investigated the influence of four different pre-treatments (immersion in solutions of citric acid, ascorbic acid, tartaric acid, and sucrose at different concentrations, 0.1%, 1% and 2%) on the anthocyanin composition of pink flowers, and they suggested that the tartaric acid solution (2%) preserved the highest amount of anthocyanins (14.78 ± 0.19 mg/g) in the sample after hot air drying. Moreover, Trivellini et al. [80] reported that salt stress (exposure to 200 mM NaCl for 28 days) was able to directly affect the content of anthocyanins present in *Hibiscus rosa-sinensis* flowers, mainly the amounts of cyanidin-3-*O*-sophoroside derivatives.

##### Chalcones

Chalcones are compounds belonging to the flavonoid class, characterized by having two aromatic rings linked by three carbons, one carbonyl and two α, β-insaturated carbons, and they are mostly abundant in plants of the Leguminosae family [81]. They are also found in Compositae and Moraceae species, being present in fruit, vegetables, and in flowers and they are able to confer yellow pigmentation, especially in the petals of some plants of medicinal use, also assisting in attracting pollinators, such as birds and insects [82]. Commonly known as open-chain flavonoids, chalcones are represented mainly by phloridzin, arbutin, phloretin, and chalconaringenin derivatives and are characterized by the absence of a ‘C-ring’ in their structural arrangement [50].

Chalcones were detected in yellow pigments of the *Coreopsis* spp. flowers [83] and are considered to be the pigments responsible for the intense yellow coloration of *Dianthus caryophyllus* L. (chalcone 2′-*O*-glucoside) and *Dahlia variabilis* (Willd.) Desf. flowers (butein and isoliquiritigenin) [84,85]. Li et al. [86], in their studies, reported that the edible flowers of *Malus pumila* Mill. present phlorizin and phloretin as the main derivatives of dihydrochalcones in their chemical composition. Other studies have reported the presence of these metabolites in the species of *Coreopsis lanceolata* L. [87] and *Coreopsis tinctoria* Nutt. [88], while Pires et al. [17] detected isoliquiritigenin-dihexoside (1.57 mg/g dw); butein-4′-glucoside (Coreopsin) (0.81 mg/g dw) and isoliquiritigenin-hexoside-acetylhexoside (0.10 mg/g dw) in *Dahlia mignon*.

##### Flavanones

Flavanones, also known as 2-phenyl-chroman-4-ones, include polyphenolic compounds, such as hesperidin, naringenin, isosacuratenin, and heridictol, with a basic structure of 2,3-dihydroflavone, which lack the double bond between C2 and C3, making them chiral at the C2 position [89]. This difference in molecular orientation plays a significant role in the way flavonoids interact with biological receptors, thereby affecting their bioactive properties [90]. In edible flowers, these compounds are associated with numerous health benefits, such as anti-aging activities, mainly related to hesperetin, hesperidin, neohesperidin, and naringin derivatives content [91]. Previous studies have reported the presence of hesperetin and its derivatives in *Chrysanthemum indicum* L. (2653.7 μg/g), *Hylocereus undatus* (Haw.) Britton & Rose (2162.2 μg/g), *Prunus persica* (L.) Batsch (850.5 μg/g), *Chrysanthemum morifolium* Ramat. (748.8 μg/g) and *Gomphrena globosa* L. (143.4 μg/g) [19]. Moreover, two derivatives of naringenin compounds (naringenin-hexoside-acetylhexoside and naringenin-3-*O*-glucoside) were found in *Dahlia* spp. at 0.82 and 2.92 mg/g dw, respectively [17]. The same genus (*Dahlia* sp.) was studied by Lara-Cortés et. al. [92], who pointed out the presence of naringenin in lilac (20.1 μg/g dw), orange (6.9 μg/g dw), and purple (1.8 μg/g dw) flowers. According to Karimi et al. [93], the flavanone naringin (688.1 1 ± 0.05 µg/g DW) stood out as one of the major compounds of the flavonoids class present in *Citrus aurantium* L. flowers. The phenolic profile of five main species of the genus *Chrysanthemum* spp. were also studied, and nine flavanones were tentatively identified among numerous compounds, namely, eriodictyol-7-*O*-glucuronide (C21), eriodictyol-7-*O*-glucoside (C22), eriodictyol (C23), naringenin-7-*O*-glucuronide (C24), narigenin (C25), flavanomarein (C26), isookain (C27), hesperetin-7-*O*-glucoside (C28), and butin (C29) [94].

##### Flavones

Flavones are characterized by the existence of double bonds between C-2 and C-3 in their extrastructural arrangement, as well as by the attachment of the B ring to C-2 [95]. They are widely found in nature and represent the second largest class of flavonoids in edible flowers, their main components being luteolin, apigenin, acacetin, chrysoeriol, and their glucosides [30]. For example, they have been detected in the flowers of *Rosa rugosa* Thunb. (1.36 μg/g), *Tropaeolum majus* L. (53.6 μg/g), *Matthiola incana* (L.) R.Br. (10.4 μg/g), florists’ chrysanthemum (4.52 μg/g), *Chrysanthemum morifolium* Ramat. (3.73 μg/g) and *Dendranthema lavandulifolium* (Fischer ex Trautv.) Kitam. (2.11 μg/g) [19] and in *Dahlia* spp. [17].

Rose, peony, and dandelion are the most common edible flowers that contain flavone compounds [96]. Moreover, the total flavones present in *Rhododendron simsii* Planch flowers constitute the major portion of the flavonoids present in its extracts [97].

##### Flavonols

Flavonols are known as the alcoholic by-product of certain flavones due to the hydroxyl group located at position 3 on the C ring, also referred to as 3-hydroxy-2- phenylchromen-4-one due to their structural similarity to flavones; these compounds are widely found in fruits, green vegetables, beverages and medicinal herbs [98,99]. Despite their similarity to flavones and being actually proanthocyanin building blocks, flavonoids are the class of metabolites most notably represented by quercitin and kaempferol that are frequently detected in edible flowers [17,30,50].

Flavonols derivatives have been observed in several ornamental plants, such as the case of kaempferol detected in *Rhododendron indicum* var. *simsii* (Planch.) Maxim. (138.5 μg/g), *Rosa centifolia* L. (12.2 μg/g), *Rosa gallica* L. (138.3 μg/g), *Bauhinia variegata* L. (91.1 μg/g), *Paeonia* × *suffruticosa* Andrews (69.0 μg/g), *Coreopsis tinctoria* Nutt. (40.9 μg/g), *Styphnolobium japonicum* (L.) Schott (38.2 μg/g), *Nymphaea nouchali* Burm.f. (28.9 μg/g) and *Matthiola incana* (L.) R.Br. (27.0 μg/g) [19]; quercetin and kaempferol derivatives in *Calendula officinalis* L. flower [100]; and quercitin (0.4 μg/mL) in *Agave durangensis* Gentry flowers [60]. Rutin was also the main compound found by Loizzo et al. [59] in the edible flowers of *Robinia pseudoacacia* L. (28.4 mg/g of extract), *Hedysarum coronarium* L. (28.2 mg/g of extract), *Sambucus nigra* L. (23.7 mg/g of extract), followed by quercitin, which was also present in *Sambucus nigra* L. (23.6 mg/g of extract), *Hedysarum coronarium* L. (8.0 mg/g of extract) and *Capparis spinosa* L. (5.8 mg/g of extract), and kaempferol, myricetin and luteolin values which were also detected. The rutin and quercitin are also prominent in some edible flower species, being one of the main flavonols found in the petals of *Malus pumila* Mill. [86], and *Citrus aurantium* L. (rutin, 362.8 ± 0.02 µg/g DW; quercetin, 185.37 ± 0.11 µg/g DW) [93].

Contents of merecitin, quercitin and kaempferol were reported in *Tagetes erecta* L. species (54.81, 13.57 and 83.42 mg/100 g dw, respectively), *Cosmos sulphureus* Cav. (59.99, 9.45 and 25.6 mg/100 g dw, respectively), *Antigonon leptopus* Hook. & Arn. (47.54, 11.08 and 75.86 mg/100 g dw, respectively) and *Bougainvillea glabra* Chosy. (61.52, 14.17 and 87.18 mg/100 g dw, respectively) [101]. Finally, derivatives of flavonols, such as quercitin and kaempferol were identified in four distinct flowers, namely *Dahlia mignon* L., *Rosa gallica* L., *Cyanus segetum* Hill and *Calendula officinalis* L. by Pires et al. [17].

##### Flavanols

Flavanols are also known as catechins and they have two chiral carbons (C2 and C3) due to the lack of double bond between C2 and C3 and carbonyl in ring C [102]. They are present in various plant parts, while they are the main constituent of *Camellia sinensis* and *C. assumica* [102,103]. Moreover, according to Yang et al. [104], the flower extracts of *Camelia nitidissima* Chi are a rich source of catechin and derivatives. The flowers of buckwheat (*Fagopyrum Esculentum* Moench) also contain the highest amounts of catechin compared to other plant parts, while *Cucurbita pepo* L. [102] and highbush blueberry (*Vaccinium angustifolium* L. [105]) flowers are also a good source of (+)-catechin and (−)-epicatechin. Krzymińska et al. [49] reported a varied content of catechins in various *Tulipa gesneriana* cultivars under different cultivation systems. In the recent study by de Morais et al. [106], the edible flowers of eight species with different colors (mini rose (*Rosa chinensis* Jacq.), torenia [*Torenia fournieri* (F.) Lind.], mini daisy (*Bellis annua* L.), clitoria (*Clitoria ternatea* L.), cosmos (*Cosmos sulphureus* Cav.), cravine (*Dianthus chinensis* L.), begonia (*Begonia* × *tuberhybrida* Voss.) and tagete (*Tagetes patula* L.) were tested and four flavanols were detected, namely catechin, epicatechin, epicatechin galate, and epigallocatechin galate. Moreover, Liang et al. [107] suggested that (−)-epicatechin was the most abundant phenolic compound in flower buds and petals of *Lilium pumilum*, while Li et al. [108] reported that catechin and epicatechin were among the most abundant phenolic compounds in the edible flowers of several species.

### 2.2. Carotenoids

Within the class of plant pigments, carotenoids are defined as fat-soluble metabolites, synthesized by photosynthetic organisms, often found in vegetables, fruit, and flowers [114,115]. Among their numerous functions, carotenoids are responsible for giving vibrant colors in shades of yellow, orange, and red, as well as for acting directly on the photoprotective system of plants [116,117,118]. In terms of classification, carotenoids are divided into two main families, namely carotenes (formed only by carbon and hydrogen, such as α-carotene, β-carotene, and lycopene) and xanthophylls (oxidized carotenes, such as lutein, zeaxanthin, and astaxanthin) [32]. Currently, approximately 650 different types of carotenoids are described in nature, out of which 100 are frequently found in the human diet [119]. Consumption of these compounds is extremely important to maintain human health and well-being and they are introduced through the consumption of food products or supplements [9,120,121].

Carotenoids are found in various parts of plants [122]. Specifically in flowers (Table 3), they are present in sepals, pollen, anthers, stamens, and petals [32]. Furthermore, it should be noted that carotenoids are key compounds in flowers, since they are responsible for enhancing the coloring of their petals, as well as promoting the functionality of their products [117,119,123]. In general, antioxidant activity stands out as the main bioactive function of carotenoids [124,125]. However, specific carotenoids are able to perform other functions through additional mechanisms, such as β-carotene that is converted into vitamin A, or some xanthophylls, such as lutein and zeaxanthin, that protect human vision by absorbing specific wavelengths of light [126]. Despite the diversity of carotenoids present in nature, the yellow xanthophylls (lutein, β-cryptoxanthin, and zeaxanthin), and epoxide xanthophylls (violaxanthin, antheraxanthin, neoxanthin) are the main carotenoids found in the flowers [127].

Several studies on the amount of total carotenoids have been reported in the literature. For example, the bright color presented by the petals of fresh pumpkin flowers was justified by the presence of high amounts of carotenoids [76]. In addition, the maturity status of *Aloe vera* flowers was studied by Martínez-Sánchez et al. [128] who verified higher contents of carotenoids (α-carotene, β-carotene, β-cryptoxanthin, zeaxanthin, and lycopene) in the younger flowers. A study with Mexican cacti edible flowers, reported the presence of red carotenoids (capsanthin and capsorubin) as the most expressive compounds in the flowers of *Opuntia oligacantha* (0.16 mg/g dw) and Echinocereus cinerascens (0.24 mg/g dw), while the yellow carotenoids (β-carotene, β-cryptoxanthin, and zeaxanthin) were detected only in the flowers of *Opuntia matudae* (0.02 mg/g dw) [54]. 

A comparative study with three species of *Dendrobium* spp. (white, light yellow, and golden yellow flowers) described the presence of eight carotenoids, namely β-carotene, α-cryptoxanthin, β-cryptoxanthin, violaxanthin, lutein, antheraxanthin, zeaxanthin, and lutein-5,6-epoxide, while *Dendrobium thyrsiflorum* B. S. Williams (golden yellow) stood out for containing the highest content of carotenoids (3810.89 μg/g) when compared to the other two species [129]. In turn, a study with golden flowers of *Lonicera japonica* Thunb. described the presence of 14 carotenoids with α-carotene, γ-carotene, and zeaxanthin being the most abundant ones [130].

The main classes of carotenoids are presented in the following sections.

#### 2.2.1. Carotenes

Carotenes are one of the first intermediates in the synthesis of carotenoids; being present in all photosynthetic organisms, they are highly abundant in our diet and accumulate in human skin, where they play a vital role in protecting skin against ultraviolet light and fighting aging [131]. *β*-carotene and lycopene are the main derivatives of the carotenes often present in intensively colored vegetables. *β*-carotene is considered a liposoluble colorant, which is sensitive to light and oxygen, being directly responsible for protection against cardiac diseases, some types of cancer, and in the oxidation of LDL-cholesterol, while lycopene is portrayed as one of the best biological suppressors in the fight against free radicals, acting directly against prostate, lung, and ovarian cancer [132]. Fernandes et al. [133], stated that although carotenes are minor in edible flowers (compared to xanthophylls), some of their derivatives (lycopene and β-carotene) are frequently cited in scientific reports due to their bioactive properties.

Eight cultivars of *Rosa hybrida* L. with different colors (white, yellow, red and pink) were studied by Yeon and Kim [134] and the results obtained presented remarkably large amounts of β-carotenes in the yellow flowers (7.62 μg/g fw in “Good time” cultivar and 4.40 μg/g fw in “Penny Lane” cultivar). Moreover, a study aiming to characterize the chemical profile of cultivars of the *Tagetes* genus (*T. erecta* L. and *T. patula* L.) highlighted the presence of seven carotenoids, four of which were derived from carotenes, namely α-carotene, β-carotene, 9 -cis-β-carotene, and 13-cis-β-carotene [116]. A literature review study on the phenolic content of five ”calendula” flower cultivars (*Calendula officinalis*, *Tagetes erecta*, *Tagetes patula*, *Tagetes lucida*, and *Tagetes tenuifolia*) highlighted *β*-carotene, *ζ*-carotene, and lycopene as the main derivatives of the present carotenes [135].

#### 2.2.2. Xanthophylls

The word “xantophylls” derives from the combination of the Greek terms xanthos (yellow) and phyllon (leaf). Xanthophylls are described as the product of oxidation of carotenoids, being responsible for giving yellowish coloration to plant tissues, and comprise the largest class of carotenoids present in nature [136]. Xanthophylls, such as lutein and zeaxanthin, play supportive roles in human vision and also help in reducing eye fatigue, muscle degeneration, and cataracts [137].

In nature, xanthophylls are often found in fruit and flowers in their esterified form by fatty acids [138,139]. The esterification process promotes the accumulation of xanthophylls in plant tissues, and consequently results in a more intense yellow coloring of the flower petals [140]. Within the xanthophyll class, lutein and zeaxanthin are mostly found in flowers, but other derivatives have been also detected in smaller proportions, namely flavoxanthin, violaxanthin, auroxanthin, antheraxanthin, and neoxanthin [32].

The flowers of Tagetes *eracta* L. are considered an excellent sources of carotenoids, mainly because they contain high amounts of lutein [141]. Lutein was also present in high proportions in chloroform extracts of *Senna bicapsularis* (L.) Roxb. (1217.2 μg/g) flowers [142]. Similarly, a review study performed by Fernandes et al. [32] highlighted lutein as the main compound found in the flowers of *Dendranthema grandiflorum* (Ramat.) Kitam. (11.78–307.22 μg/g), *Antirrhinum majus* L. (14.1 μg/g), *Tropaeolum majus* L. (350–450 μg/g), *Tagetes eracta* L. (1062 μg/g), *Tropaeolum pentaphyllum* Lam. (243.23 μg/g) and *Viola* × *wittrockiana* Gams. (51.1 μg/g).

### 2.3. Alkaloids

Alkaloids are secondary metabolites present in great abundance in nature and more than 12,000 compounds have been identified [159]. They are characterized by their bitter taste and can be found in glycosylated, methylated, and hydroxylated forms, in which nitrogen (N) is present as a characteristic element of their structure [160]. Thus, alkaloids can be classified according to their structural characteristics and their biosynthesis process, being named as true alkaloids, pseudoalkaloids, protoalkaloids [161].

Alkaloids were detected in various species with edible flowers such as Tussilago farfara L. [162,163], *Tecomella undulata* (Sm.) Seem. [164], *Erythrina mulungu* Benth. [165], and *Datura metel* L. [166] (Table 4). Through chromatographic analysis of *Sophora viciifolia* Hance extracts it was possible to identify eight quinolizidine alkaloids [167], while in samples of flowers from *Tabernaemontana divaricata* (L.) R.Br. ex Roem. & Schult. three indolic alkaloids, namely voacangine, catharanthine, and *O*-acetyl vallesamine, were also identified [168]. Moreover, alkaloids were found in pumpkin flowers (*Cucurbita maxima* Duchesne), in which the results expressed in terms of atropine were presented both for the aqueous extract (2.29 µg/mL) and the methanolic extract (1.76 µg/mL) [76].

#### 2.3.1. Betalains

Among the numerous existing alkaloids, betalains are responsible for conferring color to a range of plant organs such as fruit and flowers [169]. By definition, betalains are water-soluble pigments synthesized from the amino acid tyrosine [170]. They are antagonistic to anthocyanins, since both compounds have not been found simultaneously in plant tissues to date [171]. However, despite their accumulation in plant parts of various species, betalains are mostly restricted to plants of the Caryophyllales order [172]. In terms of classification, they are divided into two main groups; namely, the class of betacyanins derived from betanidin and presenting red and violet coloration, and betaxanthins, arising from the condensation of betalamic acid with α-amino acids or amines, which present coloration between the shades of orange and yellow [173,174].

There have been several scientific efforts to discover betalain compounds and their derivatives in flowers. For example, Lavanya et al. [175] studied the influence of seven solvents (water (T1), hot water (T2), 50% methanol (T3), 100% methanol (T4), 50% ethanol (T5), 100% ethanol (T6), and acetone (T7)) on the extraction of total betalain derivatives, betacyanins and betaxanthin, in dehydrated and fresh flowers of *Bougainvillea spectabilis* Willd. and *Celosia argentea* L. Among the results obtained, the aqueous extract (T1) showed higher yields for total betalain (24.05 ± 0.16 mg/mL), betacyanin (46.66 ± 0.04 mg/mL), and betaxanthin (54.70 ± 0.60 mg/mL) in the dehydrated flowers of *B. spectabilis*. Moreover, the yields for total betalain (25.75 ± 0.09 mg/mL), betacyanin (30.62 ± 0.32 mg/mL), and betaxanthin (31.16 ± 0.75 mg/mL) were higher in the dehydrated flowers of *C. argentea* [175].

In the study about the betalain content present in flowers of *Gomphrena globosa* L., a value of 557 mg/kg fresh weight was observed for the purple petals, while traces of betaxanthin were found for red and orange flowers of the same species in which the respective values were 75 and 45 mg/kg; however, the betacyanin content of the red petals was higher than their betaxathin content, but not a similar trend occurred in orange flowers [176]. Additionally, in the study on the betalain profile of *Schlumbergera bridgesii* (Lem.) Loefgr. cactus flowers, 14 distinct pigments were discovered, but only 7 betalain derivatives were identified, while the main betalains were vulgaxanthin I, betalamic acid, betanin, and phyllocactin (6′-*O*-malonylbetanin) [177].

##### Betaxanthins

Betaxanthins are yellowish pigments of the betalains group, resulting from the condensation of betalamic acid with amines or amino acids. In nature, the yellow tint of betalain compounds promotes the attraction of pollinating insects and plays an important part in the life cycle of flowers [178]. Previous literature studies indicated that the first betaxanthin identified in flowers were vulgaxanthin I in specimens of *Beta vulgaris* L., followed by dopaxanthin and miraxanthin V, which were found in flowers of *Lampranthus* productus N.E. Br. [179,180]. However, despite the existence of betaxanthin derivatives in flowers, there are few scientific studies that report the quantification and identification of these compounds, making them scarce when compared to other compounds present in flowers.

##### Betacyanins

Originating from the Greek kyaneos (blue), the term betacyanin is related to the color properties of its compounds, which promote pigmentation in shades of intense violet in plants [181]. The presence of betacyanins is mainly limited to the order *Caryophyllales* spp., which is composed of 35 plant families, including approximately 10,000 distinct species [181]. Although betacyanins have similar coloration to anthocyanins, in general they have a more pronounced coloring potential, more pH stability, and can be used in alkaline food matrices [182,183,184].

Several studies report the use of edible flowers rich in betacyanins as natural colorant alternatives (Table 4). For example, the American Indian Hopi community colored cornbread with extract of *Amaranthus cruentus* L. flower, consumed in their religious rites [181]. Betacyanins have also been reported in *Amaranthus tricolor* L. [185], *Gomphrena globosa* L. [184,185,186] and *Bougainvillea glabra* Choisy [187]. Moreover, a recent study evaluated the incremental use of pigments extracted from *Gomphrena globosa* L. in biscuit production and also identified the profile of the following betacyanins: gomphrenin I, isogomphrenin I, cis-Isomer of gomphrenin III, cis-Isomer of isogomphrenin III, gomphrenin II, gomphrenin III, and isogomphrenin III [183]. In addition, betalains were detected in *Amaranthus hypochondriacus* L. and *Amaranthus caudatus* L. [182].

### 2.4. Other Compounds

Other compounds that have been detected in the flowers of various species include sterols and terpenoids, as in the case of common marigold (*Calendula officinalis*) where sterols and steroids, free and esterified triterpenic alcohols, and triterpenic glycosides were identified [135]. Moreover, Veigas et al. [146] reported the presence of sterols in floral petals of *Delonix regia* (Hook.) Raf. Other examples include the species of *Cleistocalyx operculatus* and *Rumex vesicarius* [189,190], or *Punica granatum* L. flowers which contain oleanolic and ursolic acids [191]. However, it has to be mentioned that usually sterols are present in high amounts in seed oils compared to flower tissues [192,193]. Regarding the terpenoids, they have been detected in flowers of wild edible plants such as *Shorea roxburghii* G. Don and *Viburnum inopinatum* Craib [194] or in *Musella lasiocarpa* [195] and *Phlogacanthus thyrsiformis* [196] flowers. Paeonia suffruticosa (tree peony), which is widely used in Chinese medicine, also contains high amounts of monoterpene glycosides [197], while *Calotropis gigantea* flowers contain calotroposid A [198].

## 3. Toxic or Poisonous Compounds

Despite the use of edible flowers for culinary purposes throughout the centuries, the flowers may contain unsafe compounds which could have adverse effects on human health when digested [199]. However, the common domestic cooking processes based on the traditional knowledge usually eliminates these compounds or diminishes their content below the safety thresholds [200]. Moreover, the use of edible flowers as garnishment in various dishes minimizes the risk of high intakes and serious toxic effects. The recent advances in analytical chemistry has allowed the identification of new compounds and the evaluation of their toxic effects on humans [201]. These compounds include trypsin inhibitors, alkaloids, hemagglutinins, cyanogenic glucosides, and oxalic acid among others [6,20]. A recent review has listed various ornamental species that contain toxic, poisonous or antinutritional factors, such as glykosides, alkaloids, terpenoids, terpenes, and inhibitory enzymes [15]. Therefore, considering the numerous species that are commonly used for their edible flowers and the genotypic differences between the various cultivars within the same species, it is suggested that detailed analysis of the chemical composition is performed to identify potential toxic compounds or food allergens [199]. Moreover, the source of edible flowers is also very important since the irrational use of agrochemicals in commercial cultivation systems could increase the risk from high contents of unsafe compounds, while contamination from environmental factors (e.g., heavy metals in polluted soils) or external impurities should be also considered for safety reasons and legislations [202]. The increasing interest for food products that include edible flowers has created a niche market whose demand has to be fulfilled through the commercial cultivation of species aiming to harvest flowers as the final product [20].

Another issue regarding the safe use of edible flowers is that, considering the local or regional interest for many species, the available information about the precautions and the processing that may be needed prior to consumption is limited to languages other than English; therefore, all this scattered knowledge should be gathered and updated by scientists and provided in widely accepted languages [203]. Moreover, the same species, especially the wild ones, may have several common names, while several species with different chemical profiles may be known under the same common name, which could result in misidentification [204]. Therefore, it is of critical importance to analyze the chemical composition of new genotypes, even for well-established species, before suggesting them for edible purposes. 

## 4. Conclusions and Future Remarks

Edible flowers are a common ingredient in traditional and gourmet dishes, with great potential in contributing to the improvement of the aesthetic and nutritional properties of food products. Current market trends and consumer needs have increased the interest in integrating flowers in dietary patterns through novel functional and healthy foods or through the reinvention of traditional recipes. The present review provides useful information regarding the chemical composition and the main groups of chemical compounds that are present in the flowers of the most common species. It also helps to identify potentially toxic compounds in certain species that need further consideration before introducing or suggesting their culinary uses. However, considering the numerous species commonly used as edible flowers and that only a small portion of them has been thoroughly studied in terms of their chemical composition and health safety, further research is needed in order to establish regulations regarding the safe consumption not only of novel species that occasionally are introduced in culinary arts but also of traditional species where a gap of knowledge still exists. Moreover, future research needs to focus on the bioactive properties of specific compounds that edible flowers contain, as well as on their bioavailability and bioaccessibility after domestic cooking processes.

## Figures and Tables

**Table 1:** The main phenolic acids commonly found in edible flowers.

Class	Compound	Species	Reference
Hydroxybenzoic Acids	Vanillic acid	*Tulipa gesneriana* L.	[49]
	Ellagic acid	*Allium ursinum* L.	[44]
		*Bellis Perennis* L.	
		*Cyanus segetum* Hill	
		*Cichorium intybus* L.	
		*Dianthus carthusianorum* L.	
		*Dianthus pavonius* Tausch	
		*Erythronium dens-canis* L.	
		*Geranium sylvaticum* L.	
		*Lavandula angustifolia* Mill.	
		*Leucanthemum vulgare* (Vaill.) Lam.	
		*Mentha aquatica* L.	
		*Paeonia officinalis* L.	
		*Primula veris* L.	
		*Primula vulgaris* Huds.	
		*Robinia pseudoacacia* L.	
		*Rosa canina* L.	
		*Rosa pendulina L.*	
		*Salvia pratensis* L.	
		*Sambucus nigra* L.	
		*Taraxacum campylodes* G.E.Haglund	
		*Trifolium alpinum* L.	
		*Viola odorata* L.	
Hydroxybenzoic Acids	Gallic acid	*Allium ursinum* L.	[44]
		*Borago officinalis* L.	
		*Bougainvillea glabra* Choisy	
		*Cichorium intybus* L.	
		*Dianthus carthusianorum* L.	
		*Lavandula angustifolia* Mill.	[48]
		*Paeonia officinalis* L.	[44]
		*Primula veris* L.	
		*Rosa canina* L.	
		*Salvia pratensis* L.	
		*Sambucus nigra* L.	
		*Tulipa gesneriana* L.	[49]
	Syringic acid	*Tulipa gesneriana* L.	[49]
	2,5-Hydroxybenzoic acid	*Tulipa gesneriana* L.	[49]
	*p*-Hydroxybenzoic acid	*Abelmoschus manihot* (L.) Medik.	[39]
		*Calycanthus floridus* L.	[19]
		*Canna indica* L.	
		*Cymbidium sinense* (Jacks.) Willd.	
		*Dianthus caryophyllus* L.	
		*Magnolia denudata* Desr	
		*Tulipa gesneriana* L.	[47]
Hydroxycinnamic Acids	*p*-Coumaric acid	*Acmella oleracea* (L.) RKJansen	[47]
		*Amaranthus hypochondriacus* L.	
		*Hibiscus roseus* Thore	[43]
		*Tropaeolum majus* L.	[47]
		*Tulipa gesneriana* L.	[47]
	*p*-Coumaric acid hexoside	*Cyanus segetum* Hill	[17]
Hydroxycinnamic Acids	Coumaric acid	*Allium ursinum* L.	[44]
		*Cichorium intybus* L.	
		*Erythronium dens-canis* L.	
		*Geranium sylvaticum* L.	
		*Mentha aquatica* L.	
		*Sambucus nigra* L.	
		*Trifolium alpinum* L.	
		*Tropaeolum majus* L.	
		*Viola odorata* L.	
	Ferulic acid	*Acmella oleracea* (L.) RKJansen	[47]
		*Amaranthus hypochondriacus* L.	
		*Allium ursinum* L.	[44]
		*Erythronium dens-canis* L.	
		*Hibiscus roseus* Thore	[43]
		*Lavandula angustifolia* Mill.	[48]
		*Paeonia officinalis* L.	[44]
		*Primula veris* L.	
		*Primula vulgaris* Huds.	
		*Tropaeolum majus* L.	[47]
		*Trifolium alpinum* L.	[44]
		*Tulipa gesneriana* L.	[49]
		*Viola odorata* L.	[44]
	Sinapic acid	*Tulipa gesneriana* L.	[49]
		*Acmella oleracea* (L.) RKJansen	[47]
		*Calendula officinalis* L.	[17]
Hydroxycinnamic Acids	5-*O*-Caffeoylquinic acid(chlorogenic acid)	*Cichorium intybus* L.	[44]
		*Cyanus segetum* Hill	[17]
		*Cyanus segetum* Hill	
		*Dahlia mignon*	
		*Erythronium dens-canis* L.	[44]
		*Geranium sylvaticum* L.	
		*Hibiscus roseus* Thore	[43]
		*Lavandula angustifolia* Mill.	[44]
		*Mentha aquatica* L.	
		*Rosa canina* L.	
		*Taraxacum campylodes* G.E.Haglund	
		*Tropaeolum majus* L.	
		*Tropaeolum majus* L.	[47]
		*Tulipa gesneriana* L.	[49]
	Caffeic acid	*Bellis Perennis* L.	[44]
		*Cyanus segetum* Hill	
		*Dianthus carthusianorum* L.	
		*Erythronium dens-canis* L.	
		*Lavandula angustifolia* Mill.	
		*Lavandula angustifolia* Mill.	[48]
		*Mentha aquatica* L.	[44]
		*Paeonia officinalis* L.	
		*Primula veris* L.	
		*Primula vulgaris* Huds.	
		*Sambucus nigra* L.	
		*Taraxacum campylodes* G.E.Haglund	
		*Tropaeolum majus* L.	
			[47]
		*Tulipa gesneriana* L.	[49]
		*Viola odorata* L.	[44]
	Caffeic acid hexoside	*Calendula officinalis* L.	[17]
		*Cyanus segetum* Hill	
	Rosmarinic acid	*Lavandula angustifolia* Mill.	[48]

**Table 2:** The main flavonoids commonly found in edible flowers.

Class	Compounds	Species	References
Anthocyanin	Delphinidin-3-*O*-glucoside	*Crocus sativus* L.	[109]
		*Nelumbo nucifera* Gaertn.	[110]
		*Paeonia × suffruticosa* Andrews	[19]
		*Robinia hispida* L.	[73]
	Delphinidin-3-*O*-sambubioside	*Hibiscus sabdariffa* L.	[111]
	Delphinidin-3-*O*-glucoside		
	Delphinidin-3,7-*O*-diglucoside	*Crocus sativus* L.	[109]
	Petunidin-3,7-*O*-diglucoside		
	Petunidin-3-*O*-glucoside	*Crocus sativus* L.	[109]
		*Nelumbo nucifera* Gaertn.	[110]
	Peonidin-3-*O*-glucoside	*Coreopsis tinctoria* Nutt.	[19]
		*Nelumbo nucifera* Gaertn.	[110]
		*Robinia hispida* L.	[73]
	Peonidin-*O*-acetylhexoside-*O*-*p*-coumaroylhexoside	*Impatiens balsamina* L.	[28]
		*Impatiens walleriana* Hook. f.	[78]
		*Impatiens walleriana* Hook. f.	
	Pelargonidin 3,5-di-*O*-glucoside	*Rosa hybrida* L.	[9]
	Pelargonidin-3-*O*-glucoside	*Robinia hispida* L.	[73]
	Pelargonidin-*O*-dihexoside	*Impatiens balsamina* L.	[28]
	Pelargonidin-*O*-hexoside-*O*-acetylhexoside		
	Pelargonidin-*O*-hexoside-*O*-desoxihexosil-hexoside		
	Pelargonidin-*O*-*p*-coumaroil-hexoside-*O*-acetyl-hexoside		
	Pelargonidin-*O*-*p*-coumaroil-hexoside-*O*-acetyl-hexoside	*Impatiens walleriana* Hook. f.	[78]
	Pelargonidin-*O*-hexosídeo-*O*-desoxihexoside-hexoside		[78]
	Malvidin-3-*O*-glucoside	*Robinia hispida* L.	[73]
		*Nelumbo nucifera* Gaertn.	[110]
	Malvidin-*O*-glucoside	*Crocus sativus* L.	[109]
	Malvidin-*O*-coumaroylhexoside-*O*-hexoside	*Impatiens balsamina* L.	[28]
	Malvidin-*O*-coumaroylhexoside		
	Malvidin-*O*-acetylhexoside-*O*-coumaroylhexoside		
	Malvidin-*O*-acetylhexoside-*O*-*p*-coumaroylhexoside	*Impatiens walleriana* Hook. f.	[78]
	Malvidin-3-*O*-*p*-coumaroylhexoside-*O*-hexoside		
	Cyanidin 3,5-di-*O*-glucoside	*Rosa hybrida* L.	[9]
	Cyanidin-3-*O*-sophoroside	*Coreopsis tinctoria* Nutt.	[19]
	Cyanidin-3-*O*-sambubioside	*Hibiscus sabdariffa* L.	[111]
		*Hibiscus moscheutos* L. × *Hibiscus coccineus* Walt	[75]
	Cyanidin-3-*O*-glucoside	*Bauhinia purpurea* L.	[108]
		*Bauhinia variegata* L.	[19]
		*Brunfelsia brasiliensis* (Spreng.) LBSm. & Downs em Reitz	[108]
		*Calliandra haematocephala* Hassk.	
		*Dianthus caryophyllus* L.	
		*Gerbera jamesonii* Bolus ex Hook. f.	
		*Hibiscus rosa-sinensis* L.	
			[41]
		*Hibiscus sabdariffa* L.	[111]
		*Ipomoea cairica* (L.) Sweet	[108]
		*Nelumbo nucifera* Gaertn.	[110]
		*Punica granatum* L.	[74]
		*Robinia hispida* L.	[73]
		*Rhododendron* spp. L.	[108]
	Cyanidin-3-*O*-glucoside	*Ageratum houstonianum* Mill.	[71]
		*Antirrhinum majus* L.	
		*Begonia semperflorens* Link e Otto	
		*Borago officinalis* L.	
		*Calendula officinalis* L.	
		*Clitorea ternatea* L.	[41]
		*Cucurbita maxima* Duchesne	[76]
		*Dendranthema grandiflorum* (Ramat.) Kitam.	[112]
		*Dianthus barbatus* L.	[71]
		*Fuchsia hybrida* hort. ex Siebert & Voss	
		*Hibiscus moscheutos* L. × *Hibiscus coccineus* Walt	[75]
		*Ixora coccinea* L.	[41]
		*Jatropha integerrima* Jacq.	[108]
		*Melaleuca grandiflora* Blanco	
		*Pelargonium peltatum* (L.) L’Hér	[71]
		*Petunia hybrida* E.Vilm.	
		*Punica granatum* L.	[41]
		*Tagetes erecta* L.	[71]
		*Tropaeolum majus* L.	
		*Viola* × *wittrockiana* Gams	
	Cyanidin-3-*O*-(3″-malonyl)glucoside	*Dendranthema grandiflorum* (Ramat.) Kitam.	[112]
	Cyanidin-3,5-*O*-diglucoside	*Punica granatum* L.	[74]
Chalcone	Chalcone 2′-*O*-glucoside	*Dianthus caryophyllus* L.	[85]
	Acetyl-marein	*Coreopsis tinctoria* Nutt.	[88]
	2′-hydroxy-4,4′-dimethoxychalcone	*Coreopsis lanceolata* L.	[87]
	Kukulkanin B		
	Lanceoletin		
	3,2′,4′-trihydroxy-4,3′-dimethoxychalcone		
	4′-hydroxy-3,4,2′-trimethoxychalcone		
	3′,4′-dihydroxy-3,4,2′-trimethoxychalcone		
	4,4′-dihydroxy-3′-methoxychalcone 2′-*O*-*β*-*D*-glucopyranoside		
	3,4,3′,4′-tetrahydroxychalcone 2′-*O*-*β*-*D*-glucopyranoside		
	4,3′,4′-trihydroxy-3-methoxychalcone 2′-*O*-*β*-*D*-glucopyranoside		
	3,4,4′-trihydroxy-3′-methoxychalcone 2′-*O*-*β*-*D*-glucopyranoside		
	3,4′-dihydroxy-4,3′-dimethoxychalcone 2′-*O*-*β*-*D*-glucopyranoside		
	3,4′-dihydroxy-4,3′-dimethoxychalcone 2′-*O*-*α*-*L*-rhamnopyranosyl-(1→6)-*β*-*D*-glucopyranoside		
	Butein-4′-*O*-malonylsophoroside	*Dahlia variabilis* (Willd.) Desf.	[84]
	Butein-4′-*O*-glucoside		
	Butein-4′-*O*-[2-*O*-(glucosyl)-6-*O*-(malonyl)-glucoside]		
	Butein-4′-*O*-[6-*O*-(malonyl)-glucoside]		
	Butein-4′-*O*-[2-*O*-(glucosyl)-glucoside]		
	Butein-4′-*O*-glucoside (Coreopsin)	*Dahlia mignon*	[17]
	Acetylcoreopsin		
	Isoliquiritigenin-4′-*O*-[6-*O*-(malonyl)-glucoside]	*Dahlia variabilis* (Willd.) Desf.	[84]
	Isoliquiritigenin-4′-*O*-[2-*O*-(glucosyl)-glucoside]		
	Isoliquiritigenin-4′-*O*-[6-*O*-(rhamnosyl)-glucoside])		
	Isoliquiritigenin-*O*-dihexoside	*Dahlia mignon*	[17]
	Isoliquiritigenin-*O*-hexoside-acetylhexoside		
Flavones	Hesperetin	*Chrysanthemum indicum* L.	[19]
		*Chrysanthemum morifolium* ramat	
		*Gomphrena globosa* L.	
		*Hylocereus undatus* (Haw.) Britton & Rose	
		*Prunus persica* (L.) Batsch	
	Hesperetin-*O*-pentosyl-rhamnoside	*Dahlia mignon*	[17]
	Hesperidin	*Dahlia* spp.	[92]
		*Jasminum sambac* (L.) Aiton	[113]
	Pentahydroxyflavanone-*O*-acetylhexoside-hexoside	*Dahlia mignon*	[17]
	Pentahydroxyflavanone-*O*-dihexoside		
	Eriodictyol-*O*-dihexoside		
	Eriodictyol-*O*-deoxyhexosyl-hexoside		
	Eriodictyol-*O*-hexoside		
	Eriodictyol-*O*-acetyldihexoside		
	Naringenin-*O*-hexoside-acetylhexoside		
	Naringenin-3-*O*-glucoside		
	Narigenin	*Prunus persica* (L.) Batsch	[19]
	Apigenin	*Chrysanthemum morifolium* Ramat.	[19]
		*Dendranthema lavandulifolium* (fischer ex Trautv.) Kitam.	
		*Florists chrysanthemum*	
		*Matthiola incana* (L.) R.Br.	
		*Rosa rugosa* Thunb.	
		*Tropaeolum majus* L.	
	Apigenin-*O*-hexoside	*Dahlia mignon*	[17]
	Apigenin-*O*-glucuronide-acetylhexoside	*Cyanus segetum* Hill	
	Apigenin-*O*-glucuronide	*Cyanus segetum* Hill	
	Luteolin-*O*-glucuronide	*Cyanus segetum* Hill	
	Chrysoeriol	*Chrysanthemum morifolium* Ramat.	[19]
Flavonols	Isorhamnetin-3-*O*-rhamnosylrutinoside	*Rosa gallica* L.	[17]
	Isorhamnetin-3-*O*-neohesperidoside		
	Isorhamnetin-3-*O*-rutinoside		
	Isorhamnetin-3-*O*-glucoside		
	Isorhamnetin-3-*O*-(6′′-acetyl)-glucoside		
	Taxifolin	*Cyanus segetum* Hill	[17]
	Quercetin	*Bauhinia variegata* L.	[19]
		*Coreopsis tinctoria* Nutt.	
		*Matthiola incana* (L.) R.Br.	
		*Rhododendron indicum var. simsii (Planch.) Maxim.*	
		*Rosa centifolia* L.	
		*Rosa gallica* L.	
		*Styphnolobium japonicum* (L.) Schott	
	Quercetin-3-*O*-glucoside	*Agave durangensis* Gentry	[60]
		*Rosa gallica* L.	[17]
	Quercetin-*O*-hexoside	*Calendula officinalis* L.	[100]
		*Rosa gallica* L.	[17]
	Quercetin-hexoside-acetylhexoside	*Cyanus segetum* Hill	[17]
	Quercetin-3-*O*-(6″-acetyl)-glucoside	*Calendula officinalis* L.	
		*Cyanus segetum* Hill	
	Quercetin-3-*O*-rhamnosylrutinoside	*Calendula officinalis* L.	
	Quercetin-3-*O*-rutinoside	*Calendula officinalis* L.	
		*Dahlia mignon*	
	Quercetin-*O*-deoxyhexosylhexoside	*Calendula officinalis* L.	
	Quercetin-*O*-pentoside	*Rosa gallica* L.	
	Quercetin-*O*-(*p*-coumaroyl)hexoside		
	Quercetin-*O*-glucuronide		
	Kaempferol	*Anchusa azurea* Mill.	[59]
		*Antigonon leptopus* Hook. & Arn.	[101]
		*Bauhinia variegata* L.	[19]
		*Bougainvillea glabra* Choisy	[101]
		*Coreopsis tinctoria* Nutt.	[19]
		*Matthiola incana* (L.) R.Br.	
		*Nymphaea nouchali* Burm.f.	
		*Paeonia × suffruticosa* Andrews	
		*Rhododendron indicum var. simsii (Planch.) Maxim.*	
		*Rosa centifolia* L.	
		*Rosa gallica* L.	
		*Styphnolobium japonicum* (L.) Schott	
	Kaempferol-*O*-hexoside	*Calendula officinalis* L.	[100]
	Kaempferol-3-*O*-[rhamnosyl-(1–6)-glucoside]	*Agave durangensis* Gentry	[60]
	Kaempferol-3,7-*O*-diglucoside		
	Kaempferol-*O*-acetylhexoside	*Cyanus segetum* Hill	[17]
	Kaempferol-rhamnosylrutinoside	*Calendula officinalis* L.	
	Kaempferol-3-*O*-rutinoside	*Rosa gallica* L.	
	Kaempferol-*O*-glucuronide		
	Kaempferol-3-*O*-glucoside		
	Kaempferol-*O*-pentoside		
	Kaempferol-*O*-rhamnoside		
	Kaempferol-*O*-(*p*-coumaroyl)hexoside		
	Kaempferol-*O*-pentosyl-rhamnosyl-hexoside	*Dahlia mignon*	
	Kaempferol-*O*-pentosyl-rhamnoside		
	Myricetin	*Anchusa azurea* Mill.	[59]
		*Antigonon leptopus* Hook. & Arn.	[101]
		*Bauhinia variegata* L.	[19]
		*Bougainvillea glabra* Choisy	[101]

**Table 3:** The main carotenoids commonly found in edible flowers.

Class	Compounds	Species	References
Xanthophylls	Antheraxanthin	*Gentiana lutea* L.	[143]
		*Lilium* spp. “Connecticut King” (yellow)	[144]
		*Viola wittrockiana* Gams.	[145]
	Astaxanthin	*Delonix regia* (Hook.) Raf.	[146]
	Capsanthin	*Lilium* spp. “Saija” (red)	[144]
	Cryptoxanthin	*Tagetes* spp.	[147]
	*β*-Cryptoxanthin	*Hemerocallis fulva* var. Angustifolia Baker	[148]
		*Ipomoea* spp.	[149]
		*Narcissus* L.	[150]
	Lutein	*Antirrhinum majus* L.	[145]
		*Borago officinalis* L.	[151,152]
		*Camellia japonica* L.	[152]
		*Centaurea cyanus* L.	[151,152]
		*Cosmos bipinnatus* Cav.	[153]
		*Delonix regia* (Hook.) Raf.	[146]
		*Dendranthema grandiflorum* Ramat. (Anastasia)	[112]
		*Dendranthema grandiflorum* Ramat. (Il Weol)	
		*Dendranthema grandiflorum* Ramat. (Kastelli)	
		*Dendranthema grandiflorum* Ramat. (Popcorn Ball)	
		*Dianthus chinensis* L.	[154]
		*Eustoma grandiflorum* (Raf.) Shinners	[155]
		*Gentiana lutea* L.	[143]
		*Hemerocallis disticha* Donn ex Ker Gawl.	[148]
		*Lilium* spp. “Connecticut King” (yellow)	[144]
		*Melampodium divaricatum* (Rich. Ex Rich.) DC.	[153]
		*Narcissus* spp.	[150]
		*Tagetes erecta* L.	[153,156]
		*Tagetes* spp.	[147]
		*Tropaeolum majus* L.	[157]
		*Viola wittrockiana* Gams.	[145]
		*Viola wittrockiana* Gams. (red)	[152]
		*Viola wittrockiana* Gams. (white)	
		*Viola wittrockiana* Gams. (yellow)	
	Violaxanthin	*Eustoma grandiflorum* (Raf.) Shinners	[155]
		*Gentiana lutea* L.	[143]
		*Lilium* spp. “Connecticut King” (yellow)	[144]
		*Rosa* spp. (Rosa “Sun City”)	[158]
		*Viola wittrockiana* Gams.	[145]
	(9*Z*)-Violaxanthin	*Lilium* spp. “Connecticut King” (yellow)	[144]
		*Rosa* spp.	[158]
	(*E*)-Violaxanthin	*Rosa* spp.	[158]
	Zeaxanthin	*Antirrhinum majus* L.	[145]
		*Hemerocallis disticha* Donn ex Ker Gawl.	[148]
		*Ipomoea* sp.	[149]
		*Tagetes* sp.	[147]
		*Viola wittrockiana* Gams.	[145]
	*trans*-Zeaxanthin	*Narcissus* spp.	[150]
Carotenes	*β*-Carotene	*Antirrhinum majus* L.	[145]
		*Borago officinalis* L.	[151,152]
		*Camellia japonica* L.	[152]
		*Centaurea cyanus* L.	[151,152]
		*Delonix regia* (Hook.) Raf.	[146]
		*Dendranthema grandiflorum* Ramat. (Anastasia)	[112]
		*Eustoma grandiflorum* (Raf.) Shinners	[155]
		*Gentiana lutea* L.	[143]
		*Hemerocallis disticha* Donn ex Ker Gawl.	[148]
		*Ipomoea* sp.	[149]
		*Narcissus* spp.	[150]
		*Rosa* spp.	[158]
		*Tagetes erecta* L.	[156]
		*Viola wittrockiana* Gams.	[145]
		*Viola wittrockiana* Gams. (red)	[152]
		*Viola wittrockiana* Gams. (white)	
		*Viola wittrockiana* Gams. (yellow)	
	Lycopene	*Tagetes erecta* L.	[156]

**Table 4:** Alkaloids and betacyanins found in edible flowers.

Class	Compounds	Edible Flowers	References
Alkaloids	8*α*-hydroxysophocarpine	*Sophora viciifolia* Hance	[167]
	13*β*-hydroxyoxymatrine		
	9*α*-acetoxymatrine		
	14*β*-hydroxylupanine		
	Sophocarpine		
	Oxymatrine		
	9α-hydroxymatrine		
	Lupanine		
	Atropine	*Cucurbita maxima* Duchesne	[76]
	Voacangine	*Tabernaemontana divaricata* (L.) R.Br. ex Roem. & Schult.	[168]
	Catharanthine		
	*O*-acetyl vallesamine		
	Isotussilagine	*Tussilago farfara* L.	[162]
	Senkirkine		
	(E)-methyl 4-(3-(4-hydroxyphenyl)-N-methylacrylamido) butanoate	*Datura metel* L.	[166]
	6,7-dimethyl-1-D-ribityl-quinoxaline-2,3(1H,4H)-dione-5′-*O*-*β*-D-glucopyranoside		
	2-Pyrrolidinemethanol	*Tecomella undulata* (Sm.) Seem.	[164]
	3-Amino-4-pyrazolecarbonitrile		
	3-(1-Methyl-2-pyrrolidinyl)piridine		
	2-Methyl-6-propylpiperidine		
	1-Piperidineethanol		
	4-Formyl-1,3-dihydro-1,3-dimethyl-2H-imidazole-2-thione		
	5-Acetylpyrimidine-2,4,6(1H,3H,5H)-trione		
Alkaloids	1-(1-Cyclohexen-1-yl) pyrrolidine	*Tecomella undulata* (Sm.) Seem.	[164]
	Decahydroquinoline		
	5,7-Dimethyl-1,3-diazadamantan-6-one		
	2,4-Dihydro-5-methyl-2-phenyl-3H-Pyrazol-3-one		
Betalains	Total Betalain	*Bougainvillea spectabilis* Willd.	[175]
		*Celosia argentea* L.	
	Vulgaxanthin I	*Schlumbergera bridgesii* (Lem.) Loefgr.	[177]
	Betalamic acid		
	Isophyllocactin		
	Betaxanthin	*Bougainvillea spectabilis* Willd.	[175]
		*Celosia argentea* L.	
	Betacyanin	*Bougainvillea spectabilis* Willd.	[175]
		*Celosia argentea* L.	
	Betanidin-6-*O*-*β*-glucosides	*Gomphrena globosa* L.	[186]
	Betanidin-6-*O*-(6′-*O*-trans-4-coumaroyl)-*β*-glucoside		
	Betanin	*Amaranthus caudatus* L.	[188]
		*Bougainvillea glabra* Choisy	[187]
		*Schlumbergera bridgesii* (Lem.) Loefgr.	[177]
	Amaranthine	*Amaranthus caudatus* L.	[188]
	Isoamaranthine		
	Isobetanin-6-*O*-*β*-glucosides	*Gomphrena globosa* L.	[186]
	Isobetanin	*Amaranthus caudatus* L.	[188]
	Phyllocactin	*Schlumbergera bridgesii* (Lem.) Loefgr.	[180]
	2′-Apiosyl-phyllocactin		
	Portulacaxanthin II	*Portulaca grandiflora* Hook.	
	Portulacaxanthin III		
	2-Descarboxy-betanidin	*Bougainvillea glabra* Choisy	
	3-Methoxytyramine-betaxanthin	*Celosia argentea* var. plumosa	

## Data Availability

Not applicable.
